# *Porphyromonas pasteri* and *Prevotella nanceiensis* in the sputum microbiota are associated with increased decline in lung function in individuals with cystic fibrosis

**DOI:** 10.1099/jmm.0.001481

**Published:** 2022-02-03

**Authors:** Karmel Webb, Nur Masirah M. Zain, Iain Stewart, Andrew Fogarty, Edward F. Nash, Joanna L. Whitehouse, Alan R. Smyth, Andrew K. Lilley, Alan Knox, Paul Williams, Miguel Cámara, Kenneth Bruce, Helen L. Barr

**Affiliations:** ^1^​ Division of Epidemiology and Public Health, University of Nottingham, City Hospital Campus, Nottingham, UK; ^2^​ Nottingham NIHR Biomedical Research Centre, Nottingham MRC Molecular Pathology Node, UK; ^3^​ Institute of Pharmaceutical Science, King’s College London, UK; ^4^​ Division of Respiratory Medicine, University of Nottingham, City Hospital Campus, Nottingham, UK; ^5^​ West Midlands Adult CF Centre, Heartlands Hospital, University Hospitals Birmingham NHS Foundation Trust, Birmingham, UK; ^6^​ School of Medicine, University of Nottingham, Nottingham, UK; ^7^​ National Biofilms Innovation Centre, Biodiscovery Institute, School of Life Sciences, University of Nottingham, Nottingham, UK; ^8^​ Wolfson Cystic Fibrosis Centre, Department of Respiratory Medicine, Nottingham University Hospitals NHS Trust, Nottingham, UK

**Keywords:** cystic fibrosis, microbiology, anaerobic infection

## Abstract

Although anaerobic bacteria exist in abundance in cystic fibrosis (CF) airways, their role in disease progression is poorly understood. We hypothesized that the presence and relative abundance of the most prevalent, live, anaerobic bacteria in sputum of adults with CF were associated with adverse clinical outcomes. This is the first study to prospectively investigate viable anaerobic bacteria present in the sputum microbiota and their relationship with long-term outcomes in adults with CF. We performed 16S rRNA analysis using a viability quantitative PCR technique on sputum samples obtained from a prospective cohort of 70 adults with CF and collected clinical data over an 8 year follow-up period. We examined the associations of the ten most abundant obligate anaerobic bacteria present in the sputum with annual rate of FEV_1_ change. The presence of *Porphyromonas pasteri* and *Prevotella nanceiensis* were associated with a greater annual rate of FEV_1_ change; −52.3 ml yr^−1^ (95 % CI-87.7;−16.9), –67.9 ml yr^−1^ (95 % CI-115.6;−20.1), respectively. Similarly, the relative abundance of these live organisms were associated with a greater annual rate of FEV_1_ decline of −3.7 ml yr^−1^ (95 % CI: −6.1 to −1.3, *P*=0.003) and −5.3 ml yr^−1^ (95 % CI: −8.7 to −1.9, *P*=0.002) for each log_2_ increment of abundance, respectively. The presence and relative abundance of certain anaerobes in the sputum of adults with CF are associated with a greater rate of long-term lung function decline. The pathogenicity of anaerobic bacteria in the CF airways should be confirmed with further longitudinal prospective studies with a larger cohort of participants.

## Introduction

Chronic pulmonary infection and recurrent pulmonary exacerbations are associated with both increased morbidity and mortality in cystic fibrosis (CF) [1]. Historically, conventional microbiological culture techniques have routinely isolated several distinct aerobic species, such as *Staphylococcus aureus, Haemophilus influenzae* and *Pseudomonas aeruginosa,* which have been extensively studied [2].

However, conventional culture techniques only identify a very small proportion of the bacteria present in the lung environment. In recent years, culture-independent techniques such as PCR and microbiome analysis have shown that the CF airways are complex polymicrobial environments and often contain anaerobic bacteria [3–5], which are not routinely cultured in clinical practice.

The CF airways contain mucus, which adheres to epithelial surfaces. Within these mucus plugs, are biofilms with steep oxygen gradients, which are considered to be hypoxic environments, providing a niche for anaerobic bacteria [6, 7]. However, it is currently not known whether these anaerobic bacteria play a pathophysiological role in lung damage, pulmonary exacerbations or long-term adverse outcomes in this patient population [6].

Sputum microbiome studies in CF have shown that the composition of bacterial communities remains relatively stable during exacerbations despite systemic antibiotic therapy [8, 9], and loss of species diversity is associated with reduced lung function [8, 10]. However, there are limited longitudinal data on the relationship between the sputum microbiome and long-term clinical outcomes [11]. Furthermore, whilst photoreactive dyes have been increasingly used to amplify and quantify only the bacterial cells with intact cell membranes in 16S rRNA sequencing [12], previous sputum microbiome studies in CF have not differentiated between viable and dead bacteria.

Using this viable cell technique, we investigated whether the presence and relative abundance of the ten most abundant, viable, obligate anaerobic bacterial species in CF sputum were associated with accelerated lung function decline in an 8 year follow up period.

## Methods

### Participants and study design

We analysed sputum samples obtained adults with CF who had previously participated in a biomarker study (Research Ethics Committee; 09 /H0407/1), the full details of which have been published [13]. In summary, participants were recruited at clinical stability from two UK adult CF centres between the years 2009 and 2011. Adults with CF were clinically stable at the study visit, having not experienced a pulmonary exacerbation requiring intravenous (IV) antibiotics in the preceding 4 weeks. Baseline demographic data were collected, and sputum plugs were stored for future microbiome analysis.

### Clinical data

Lung function data were obtained from the UK CF registry [14] using the highest recorded forced expiratory volume in 1 s (FEV_1_) of the preceding year. Annual data on lung function were collected from the participants from the year of recruitment to the end of the study period in 2017 or until year of death or lung transplantation.

### Sample processing and microbiome analysis

Sputum plugs were harvested for microbiome analysis with an equal volume of 0.9 % saline and stored in −80 °C freezers. Prior to DNA extraction, known weight of sputum aliquots (up to 0.2 g) were pre-treated with propidium monoazide (PMA). PMA is unable to penetrate intact cell membranes and therefore only binds to the DNA of cells with compromised cell membranes [15]. Photo-activation of PMA results in covalent DNA modification and damage. This subsequently prevents amplification via PCR. Non-PMA treated matching sputum samples were used as positive controls.

DNA extraction was conducted using GenElute Bacterial Genomic DNA Kit (Sigma-Aldrich, UK) according to the manufacturer’s instructions, incorporated with additional enzymatic and physical disruption of samples. DNA was resuspended in 50 µl of Elution Solution and quantified using a Picodrop Microlitre Spectrophotometer (GRI, UK). Phosphate-buffered solution was used as negative controls in DNA extraction and library preparation for sequencing and no amplification was detected above the lowest limit of detection (10 c.f.u. µl^−1^). Paired-end (300 bp) sequencing of 16S rRNA V3-V4 region for 75 DNA samples were then performed on the MiSeq platform (Illumina, USA). Sequence processing and analysis included quality filtering of reads, chimaera removal and assignment to Operational Taxonomic Units (OTUs) via the C9VLC pipeline and the QIIME software package (version 1.9.1, http://qiime.org/) (Eurofins, Germany).

### Statistical analysis

Yearly change in lung function was predicted for each individual using linear regression models of CF registry-recorded FEV_1_ values with their corresponding dates, and is reported as FEV_1_ ml change per year (ml yr^−1^).

The relative abundance of each OTU was initially expressed as a percentage (%) of the total number of reads in each individual. To allow comparison across the cohort, the % OTUs were transformed to c.f.u. (c.f.u. g^−1^) of sputum by multiplying the % values with live total bacterial load in each sample accordingly, as informed by quantitative PCR (qPCR) [16]. The relative abundance (c.f.u. g^−1^ of sputum) was subsequently transformed after the addition of 1 (log_2_) for statistical analyses. The ten most abundant OTUs were determined by ranking the relative abundance of the OTUs present in all participants (Table S1, available in the online version of this article). Only the taxonomic level of species were included in the analyses. Generalized linear regression models were used to analyse the presence or absence and relative abundance (log_2_) on the annual rate of FEV_1_ change. Model fit was assessed using Akaike and Bayesian information criterion and both the dependant variable (FEV_1_ decline) and model residuals were assessed and confirmed to be normally distributed.

All analyses were performed in Stata SE15 statistical software (Texas, USA), a *P*-value<0.005 was considered statistically significant following Bonferroni correction for multiple comparison of ten OTUs.

## Results

Sputum samples and data were available for 70 (93.1 %) of the 75 participants in the original study.

Sixty-eight (97.1 %) of participants were chronically colonized with *P. aeruginosa* [17]. Baseline clinical characteristics and OTU abundance are summarized in Table 1.

**Table 1.** Baseline clinical characteristics, summary of clinical data during the follow-up period and OTU abundance in each participant

Variable	Baseline (*n*=70)	
Age in years: median (range)	30.6 (17.8 to 61.5)	
Gender, males (%)	46 (65.1)	
FEV_1_ % predicted: mean (sd)	58 (±20)	
Absolute FEV_1_ in l: mean (sd)	2.2 (±0.9)	
BMI in kg m^−2^: mean (sd)	22.9 (±3.4)	
* P. aeruginosa* status at baseline: *n* (%)		
Never	0 (0)	
Free	1 (1.4)	
Intermittent	1 (1.4)	
Chronic	68(97.1)	
**Variable**	**N (%)**	**Outcome**
Followup time* (years)	70 (100)	6.8 (6.2–8.1)
Number of lung transplantation	8 (11.4)	
Number of deaths	10 (14.2)	
Rate of lung function change per year†:		
Absolute FEV_1_ (ml)	68 (97)	−53.2 (±71.4)
Percent predicted FEV_1_ (%)	68 (97)	−1.4 (±1.9)
**Variable per participant (** ***n*** **=70)**	**Range**	**Mean (SD)**
Number of reads	9748–48 504	17 458.9 (±6306.1)
Number of OTUs	5–44	22.7 (±9.6)
Number of obligate anaerobe OTUs	0–17	6.3 (±4.4)
Total abundance, c.f.u. g^−1^ of sputum	5.0×10^4^–3.9 x 10^7^	3.5×10^6^ (6.0x10^6^)
Obligate anaerobe abundance, c.f.u. g^−1^ of sputum	0–7.6 x 10^6^	6.1×10^5^ (1.3x10^6^)

*n*=number of participants with data available; SD=standard deviation, *P. aeruginosa* status of participants defined by Leeds criteria [17].

*Reported as median and interquartile range.

†Reported as mean and standard deviation.

**Fig. 1.**
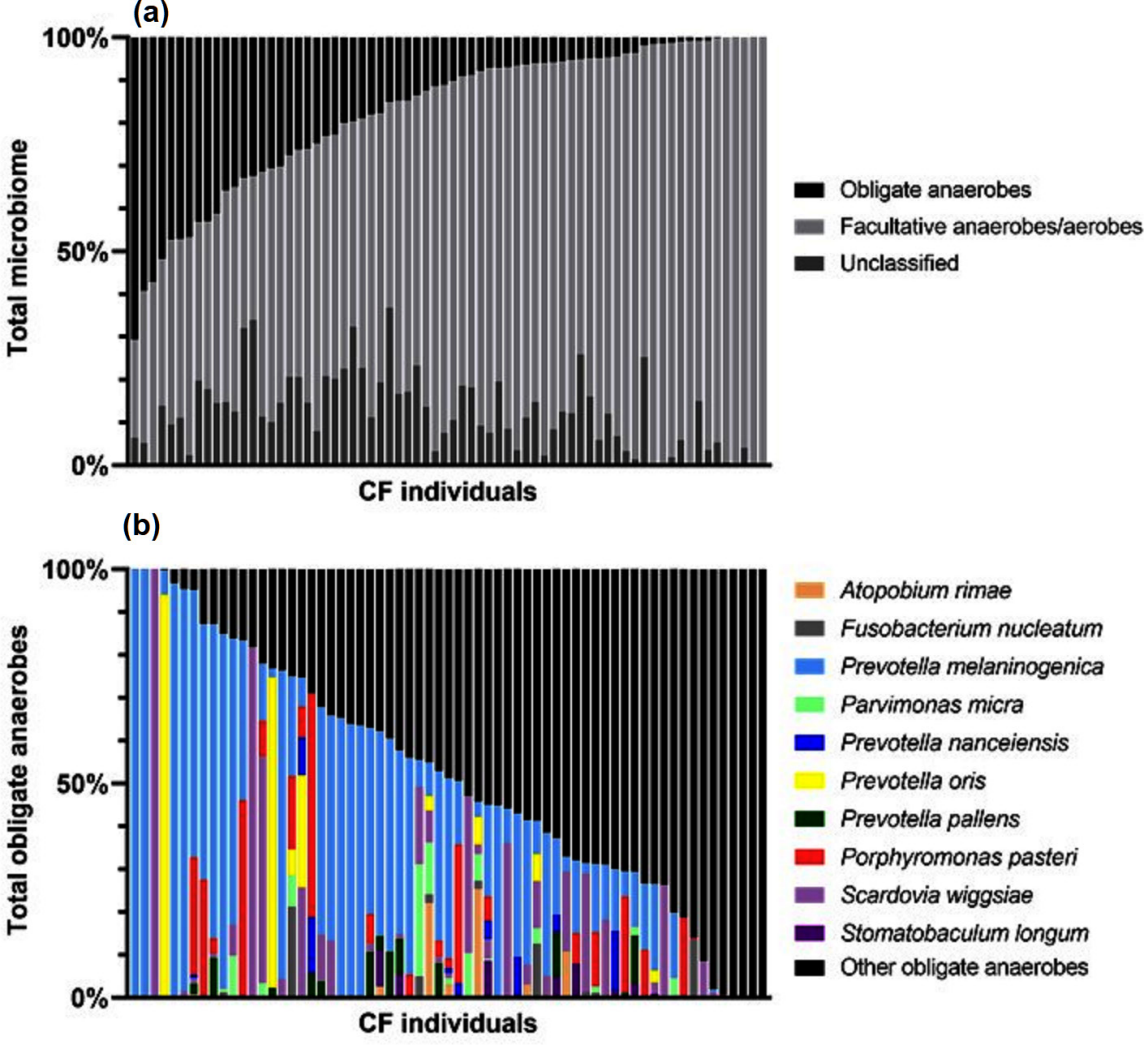
Composition of (a) total obligate anaerobe OTUs within the viable CF total microbiome at clinical stability (*n*=70) and (b) individual OTUs included in the analysis within the total obligate anaerobe group (*n*=65).

A total of 1.2 million sequencing reads were obtained from the pooled sputum of each participant (*n*=70) and assigned to 212 OTUs. The assignment of 212 OTUs was determined by best-matching reference sequences to the most specific (lowest) taxonomic level. Within this dataset, 121 OTUs were classified as facultative anaerobes or aerobes, 73 OTUs as obligate anaerobes, while 18 OTUs assigned to taxa above genera were categorized as ‘unclassified’. The lung microbiome for each CF individual varied from 5 to 44 different OTUs, with the highest richness of obligate anaerobes observed at 17 OTUs per participant (Table 1). Obligate anaerobes were present in 65/70 CF individuals, ranging from 0.1–70.6 % of the total microbiome (Fig. 1). A list of OTUs and classification are presented in Tables S1 and S2. The ten most prevalent obligate anaerobes are summarized in Table S3.

### Impact of the ten most abundant anaerobic species present in sputum on annual rate of FEV_1_ change over an 8 year follow-up period

Two of the ten anaerobic species studied were associated with lung function decline: *Porphyromonas pasteri* and *Prevotella nanceiensis* (Table 2). The effect size of annual rate of FEV_1_ decline if these viable organisms were present compared to absent was −52.3 ml yr^−1^ (95 % CI:−87.7 to −16.9, *P*=0.004) and −67.9 ml yr^−1^ (95 % CI: −115.6 to −20.1, *P*=0.005 (Table 2), respectively. Similarly, the relative abundance of live *P. pasteri* and *P. nanceiensis* was associated with a greater annual rate of FEV_1_ decline of −3.7 ml yr^−1^ (95 % CI: −6.1 to −1.3, *P*=0.003) and −5.3 ml yr^−1^ (95 % CI: −8.7 to −1.9, *P*=0.002; Table 2) for each log_2_ increment of abundance, respectively.

**Table 2.** Effect size of the presence relative to the absence and relative abundance of the ten most abundant obligate anaerobic species on annual rate of FEV_1_ (ml) decline, *N*=68

Anaerobic species	*N* (%)	Coefficient (β)±	95 % CI±	Coefficient (β)*	95 % CI*
* Prevotella melaninogenica *	50 (73.5)	24.7	−13.6; 63.0	0.4	−2.0; 2.8
* Scardovia wiggsiae *	15 (22.1)	−7.6	−43.0; 27.8	−1.8	−4.1; 0.5
* Porphyromonas pasteri *	9 (13.2)	−52.3	−**87.7; −16.9**	−3.7	−**6.1; −1.3**
* Parvimonas micra *	7 (10.3)	6.5	−34.7; 47.7	−0.7	−4.1; 2.7
* Prevotella pallens *	20 (29.4)	14.4	56.5; 27.8	−0.7	−3.8; 2.4
* Stomatobaculum longum *	9 (13.2)	11.8	−38.6; 62.2	−0.7	−4.4; 2.8
* Fusobacterium nucleatum *	8 (11.8)	35.4	14.3; 85.2	2.3	−1.7; 6.2
* Prevotella nanceiensis *	31 (45.6)	−67.9	−**115.6; −20.1**	−5.3	−**8.7; −1.9**
* Prevotella oris *	14 (20.6)	−11.2	−64.2; 41.8	−1.0	−4.4; 2.4
* Atopobium rimae *	9 (13.2)	−22.6	−78.6; 33.4	−2.0	−5.5; 1.5

*N*: number of bacteria present in sputum of participants, β: coefficient of annualized rate of FEV_1_ decline (ml yr^−1^) ±: Effect size of annual rate of FEV_1_ decline if bacterial species is present relative to absent, *: log_2_ relative abundance and annual rate of FEV_1_ decline, 95 % CI; confidence intervals. 95 % CI in bold are considered significant after Bonferroni correction (*P*=0.005).

## Discussion

This is the first study to prospectively investigate viable anaerobic bacteria present in the sputum microbiota and relationship to long-term outcomes in adults with CF. The presence and relative abundance of both *Porphyromonas pasteri* and *Prevotella nanceiensis* in a cohort predominately colonized with *Pseudomonas aeruginosa* were associated with a greater annual lung function decline over the 8 year study period.

The lower airways in CF are polymicrobial and often contain anaerobes [3–5]. These anaerobes may be the result of repeated micro aspirations of oral flora in combination with an abnormal mucociliary clearance mechanism in CF airways [18], although not all data support this hypothesis [19–21]. For example, Rogers *et al*. demonstrated dissimilarity with paired expectorated sputum and mouthwash samples from the same CF individuals [22]. Despite the high abundance of anaerobes in the CF lower airways, their pathogenicity in the CF airways is not well understood. There is much deliberation over whether obligate anaerobic bacteria, such as *Prevotella* sp., which are known to be pathogenic and to harbour antimicrobial resistance genes [23], can also interact with other bacterial pathogens, such as *P. aeruginosa,* to enhance their virulence and growth [24, 25]. Indeed, evidence suggests *P. aeruginosa,* which preferably uses aerobic respiration, undergoes anaerobiosis in the presence of nitrate in the CF airways [26]. *Prevotella* species can protect *P. aeruginosa* against the activity of ceftazidime, an anti-pseudomonal antibiotic commonly used to treat pulmonary exacerbations [27]. Our data support these observations, suggesting that *Prevotella* sp. may be associated with adverse clinical outcomes. However, our observation that *Porphyromonas* was associated with adverse clinical outcomes is not supported by other studies. Studies in children with CF found that *Porphyromonas* sp. may protect against *P. aeruginosa* colonization [28, 29] and *Porphyromonas* sp. is also less abundant during pulmonary exacerbations, thus suggesting a protective role [28]. However, these studies were both performed in children without *P. aeruginosa,* while our study was performed in adults with chronic pulmonary *P. aeruginosa* and more severe pulmonary disease. We suggest that changes in community dynamics over time, spatial heterogeneity of the lungs and differing microenvironments may account for the contrasting observations. Zemanick *et al*. found that the presence of sputum anaerobes during a pulmonary exacerbation were associated with improved lung function and less inflammation [30], although there was significant variability in anaerobes in response to antibiotics (15). This suggests that diverse anaerobic species present in the airway may have distinct pathogenic or protective roles depending on individual interactions in the lung microbiota [11, 31].

Strengths of the study include the multicentre study population who were recruited from two adult specialist CF centres. Longitudinal follow up over an 8 year period provided robust linear modelling of lung function to predict FEV_1_ outcomes. In addition, by using a novel PMA pre-treatment, we were able to give a more accurate estimate of viable anaerobic bacteria.

There are a number of limitations in this study that should be considered when interpreting these data. The enumeration of bacterial cells using the viability PCR technique has not been validated in comparison to standard culture methods for CF sputum [15]. Sputum samples were taken once at baseline only and compared with adverse clinical outcomes therefore findings should be considered as hypothesis generating. Whilst this offers novel insights, longitudinal profiling of the sputum microbiota (including aerobic and facultative anaerobic bacterial species) in a larger cohort and over multiple time points would aid our understanding further on the microbiological anaerobic community dynamics.

Our findings support a potential association of specific anaerobic species abundantly present in CF sputum with long-term lung function decline. These findings suggest that *Prevotella* and *Porphyromonas* species may contribute to lung disease and CF pathophysiology, which should be confirmed with a large, prospective cohort with regular microbiome profiling combined with enhanced validated sequencing strategies.

## Supplementary Data

Supplementary material 1
