# Supplementary material for: Porphyromonas pasteri and Prevotella nanceiensis in the sputum microbiota are associated with increased decline in lung function in individuals with cystic fibrosis
Source: J Med Microbiol. 2022 Feb 3;71(2):001481. doi: 10.1099/jmm.0.001481 (PMC8941952; doi:10.1099/jmm.0.001481)
Supplement: Supplementary material 1 [file jmm-71-1481-s001.pdf]

Supplementary materials

Porphyromonas pasteri **and** ***Prevotella nanceiensis*** **in the sputum microbiota are associated with increased decline in lung function in individuals with cystic fibrosis**

Karmel Webb[a h*], Nur Masirah M. Zain [b h*], Iain Stewart[c h] , Andrew Fogarty[a h], Edward F. Nash[d], Joanna L Whitehouse[d], Alan R. Smyth[e h], Andrew K. Lilley [b], Alan Knox [c h] Paul Williams[f], Miguel Cámara[f], Kenneth Bruce[b h], Helen L. Barr[g h]

Table 1. The summary of Operational Taxonomic Units (OTUs) in sputum microbiota of cystic fibrosis individuals at clinical stability. Obligate anaerobe OTUs were identified based on literature [1, 2] and culture collections databases (NCTC, ATCC and DSMZ).

	Operational Taxonomic Unit (OTU)	Number of individuals, n	Prevalence, %	Cumulative species abundance, $\log_2$ CFU/g of sputum
**Obligate anaerobes**	*Prevotella melaninogenica*	52	74.3	23.52
	Prevotella sp.	43	61.4	23.39
	Scardovia wiggsiae	31	44.3	21.13
	Alloprevotella Prevotella sp. oral taxon 474	22	31.4	19.81
	Porphyromonas pasteri	21	30.0	20.07
	Porphyromonas sp.	17	24.3	20.42
	Parvimonas micra	15	21.4	18.42
	Veillonella sp.	15	21.4	18.38
	Peptostreptococcus sp.	14	20.0	19.54
	Prevotella pallens	14	20.0	19.10
	Catonella sp.	11	15.7	17.87
	Atopobium sp.	10	14.3	19.83
	Fusobacterium nucleatum	9	12.9	18.04
	Mogibacterium sp.	9	12.9	16.44
	Prevotella nanceiensis	9	12.9	18.33
	Stomatobaculum longum	9	12.9	20.07
	Prevotella oris	8	11.4	21.47
	Atopobium rimae	7	10.0	20.07
	Ihubacter sp.	7	10.0	18.68
	Alloprevotella tannerae	6	8.6	16.14
	Porphyromonas endodontalis	6	8.6	17.75
	Prevotella oulorum	6	8.6	19.66
	Oribacterium sp.	5	7.1	17.75
	Peptoniphilus sp.	5	7.1	15.99
	Porphyromonas catoniae	5	7.1	16.05
	Prevotella denticola	5	7.1	18.50
	Finegoldia sp.	4	5.7	14.14
	Porphyromonas sp. C1075	4	5.7	16.43
	Prevotella histicola	4	5.7	17.29
	Tannerella forsythia	4	5.7	14.21
	Tannerella sp.	4	5.7	16.03
	Tannerella sp. oral taxon HOT-286	4	5.7	14.97
	Anaerococcus sp.	3	4.3	12.87
	Fusobacterium periodonticum	3	4.3	12.64
	Moryella indoligenes	3	4.3	19.76
	Shuttleworthia sp.	3	4.3	15.87
	Bifidobacterium sp.	2	2.9	20.31
	Dialister sp.	2	2.9	15.69
	Olsenella sp.	2	2.9	13.67
	Oribacterium parvum	2	2.9	11.81
	Peptostreptococcus anaerobius	2	2.9	16.38
	Prevotella nigrescens	2	2.9	15.10
	Prevotella salivae	2	2.9	13.67
	Solobacterium moorei	2	2.9	11.17

	Anaerococcus obesiensis	1	1.4	11.23
	Atopobium sp. DMCT15023	1	1.4	17.61
	Bacteroides fragilis	1	1.4	11.40
	Bifidobacterium breve	1	1.4	11.23
	Catonella morbi	1	1.4	12.53
	Fusobacterium necrophorum	1	1.4	12.81
	Johnsonella sp. oral taxon 166	1	1.4	14.82
	Lachnoanaerobaculum umeaense	1	1.4	16.61
	Megasphaera sp. sp4-iso-1H02x2	1	1.4	15.25
	Parvimonas sp. KA00067	1	1.4	8.88
	Peptococcus sp. oral taxon 167	1	1.4	12.97
	Porphyromonas gingivalis	1	1.4	9.97
	Prevotella conceptionensis	1	1.4	13.77
	Prevotella intermedia	1	1.4	14.05
	Prevotella shahii	1	1.4	12.26
	Prevotella sp. 8404125	1	1.4	10.89
	Prevotella sp. oral taxon 292	1	1.4	14.55
	Prevotella sp. oral taxon 299	1	1.4	11.29
	Ruminiclostridium cellobioparum	1	1.4	11.60
	Scardovia sp.	1	1.4	13.43
	Shuttleworthia sp. MSX8B	1	1.4	12.00
	Slackia sp. CM382	1	1.4	12.97
	Sneathia amnii	1	1.4	10.10
	Sneathia sanguinegens	1	1.4	11.23
	Treponema lecithinolyticum	1	1.4	15.67
	Treponema maltophilum	1	1.4	12.73
	Treponema sp. OMZ 838	1	1.4	9.88
	Veillonella atypica	1	1.4	15.18
	Veillonella parvula	1	1.4	11.89
**Facultative anaerobes/ Aerobes**	*Streptococcus sp.*	69	98.6	25.22
	Rothia sp.	61	87.1	23.39
	Pseudomonas sp.	55	78.6	23.74
	Rothia mucilaginosa	50	71.4	23.63
	Gemella sp.	47	67.1	20.31
	Actinomyces sp.	43	61.4	22.25
	Pseudomonas aeruginosa	28	40.0	20.19
	Staphylococcus sp.	22	31.4	25.69
	Actinomyces odontolyticus	18	25.7	20.93
	Staphylococcus aureus	18	25.7	23.25
	Ralstonia solanacearum	14	20.0	17.81
	Serratia sp.	14	20.0	16.75
	Streptococcus mitis	14	20.0	17.61
	Flavobacterium sp.	13	18.6	19.48
	Actinomyces sp. 1AG30-1x10	11	15.7	18.99
	Actinomyces sp. sp2-iso-aAG3x2	11	15.7	20.42
	Capnocytophaga gingivalis	11	15.7	19.44
	Granulicatella elegans	10	14.3	18.93
	Streptococcus salivarius	10	14.3	15.94
	Streptococcus sp.	10	14.3	15.80
	Streptococcus sp. 3192A	10	14.3	15.01
	Leptotrichia sp.	9	12.9	19.42

	Streptococcus pneumoniae	9	12.9	14.36
	Leptotrichia hongkongensis	8	11.4	15.39
	Capnocytophaga sputigena	8	11.4	18.84
	Mycoplasma salivarium	8	11.4	18.93
	Pseudomonas sp. CFWTS4	8	11.4	13.55
	Streptococcus mutans	8	11.4	17.87
	Actinomyces naeslundii	7	10.0	17.10
	Arthrobacter sp.	7	10.0	13.21
	Haemophilus sp.	7	10.0	18.42
	Lactobacillus sp.	7	10.0	15.72
	Neisseria sp.	7	10.0	15.61
	Actinomyces graevenitzii	6	8.6	18.68
	Capnocytophaga sp.	6	8.6	16.75
	Dysgonomonas sp. PH5-45	6	8.6	16.03
	Pseudomonas sp. 1W	6	8.6	12.26
	Pseudomonas sp. WHAS46	6	8.6	13.43
	Streptococcus anginosus	6	8.6	20.31
	Actinomyces sp. R42.11	5	7.1	15.85
	Capnocytophaga granulosa	5	7.1	19.07
	Gemella haemolysans	5	7.1	14.72
	Gemella morbillorum	5	7.1	16.10
	Lautropia sp.	5	7.1	16.99
	Pseudomonas otitidis	5	7.1	11.40
	Streptococcus sp. oral taxon 071	5	7.1	11.29
	Leptotrichia genomo sp. C1	4	5.7	14.97
	Abiotrophia defectiva	4	5.7	16.61
	Actinomyces gerencseriae	4	5.7	15.29
	Actinomyces lingnae	4	5.7	15.90
	Eubacterium sp. oral strain A35MT	4	5.7	13.55
	Haemophilus parainfluenzae	4	5.7	15.77
	Streptococcus lactarius	4	5.7	18.04
	Streptococcus peroris	4	5.7	14.92
	Achromobacter sp.	3	4.3	22.36
	Actinomyces sp.	3	4.3	16.61
	Actinomyces sp. ICM39	3	4.3	15.36
	Actinomyces sp. oral taxon 170	3	4.3	13.97
	Actinomyces sp. oral taxon 448	3	4.3	16.99
	Campylobacter concisus	3	4.3	13.77
	Gemella sp. sp2-iso-ab31	3	4.3	14.21
	Janthinobacterium sp.	3	4.3	12.53
	Pseudomonas sp. P1-5	3	4.3	12.10
	Streptococcus oralis	3	4.3	12.73
	Yokenella sp.	3	4.3	13.67
	Leptotrichia shahii	2	2.9	15.67
	Leptotrichia wadei	2	2.9	17.54
	Actinomyces sp. sp4-iso-1H01	2	2.9	20.07
	Aggregatibacter aphrophilus	2	2.9	11.97
	Campylobacter gracilis	2	2.9	16.75
	Capnocytophaga sp. S12-14	2	2.9	13.77
	Corynebacterium durum	2	2.9	13.97
	Corynebacterium sp.	2	2.9	17.38

	Enterococcus sp.	2	2.9	16.39
	Gemella sanguinis	2	2.9	13.18
	Kingella oralis	2	2.9	13.77
	Pseudomonas resinovorans	2	2.9	11.17
	Pseudomonas sp. EJ01	2	2.9	10.97
	Streptococcus constellatus	2	2.9	13.27
	Streptococcus cristatus	2	2.9	15.36
	Streptococcus massiliensis	2	2.9	11.04
	Leptotrichia hofstadii	1	1.4	9.10
	Leptotrichia massiliensis	1	1.4	11.23
	Leptotrichia sp. oral taxon 212	1	1.4	11.85
	Leptotrichia sp. PG10	1	1.4	13.43
	Achromobacter xylosoxidans	1	1.4	15.21
	Actinobaculum sp.	1	1.4	11.45
	Actinomyces oris	1	1.4	14.29
	Actinomyces sp. ChDC B246-I	1	1.4	12.62
	Actinomyces sp. ICM41	1	1.4	14.82
	Actinomyces sp. ICM54	1	1.4	14.49
	Actinomyces sp. oral strain Hal-1065	1	1.4	10.64
	Actinomyces sp. oral taxon 848	1	1.4	15.75
	Ancylomarina subtilis	1	1.4	9.20
	Bergeyella cardium	1	1.4	12.93
	Campylobacter sp.	1	1.4	15.69
	Cardiobacterium hominis	1	1.4	9.95
	Cardiobacterium valvarum	1	1.4	10.89
	Corynebacterium segmentosum	1	1.4	16.32
	Corynebacterium sp.	1	1.4	11.34
	Corynebacterium sp. oral taxon B00	1	1.4	9.08
	Enterococcus faecium	1	1.4	13.67
	Gemella asaccharolytica	1	1.4	10.10
	Haemophilus haemolyticus	1	1.4	10.81
	Haemophilus parahaemolyticus	1	1.4	14.61
	Haemophilus sp. HFH0072	1	1.4	11.23
	Haemophilus sputorum	1	1.4	9.18
	Lachnobacterium sp.	1	1.4	11.17
	Lawsonella clevelandensis	1	1.4	13.29
	Marinifilum sp. S1-C	1	1.4	8.41
	Moraxella nonliquefaciens	1	1.4	17.75
	Neisseria cinerea	1	1.4	11.89
	Nocardia sp.	1	1.4	17.10
	Ottowia sp. Marseille-P4747	1	1.4	10.55
	Rothia dentocariosa	1	1.4	13.29
	Staphylococcus sp. KSUM 23	1	1.4	15.10
	Streptobacillus hongkongensis	1	1.4	12.55
	Streptococcus australis	1	1.4	6.91
	Streptococcus sp. C206	1	1.4	8.68
	Streptococcus sp. oral taxon G62	1	1.4	9.85
	Sulfurihydrogenibium sp. T-7	1	1.4	10.89

**Unclassified**	*Lactobacillales (order)*	57	81.4	22.49
	Streptococcaceae (family)	53	75.7	21.93
	Bacteria (Kingdom)	52	74.3	23.16
	Actinobacteria (phylum)	34	48.6	22.78
	Firmicutes (phylum)	31	44.3	19.35
	Actinobacteria (class)	28	40.0	20.42
	Clostridiales (order)	21	30.0	18.29
	Enterobacterales (order)	5	7.1	13.09
	Neisseriaceae (family)	5	7.1	13.67
	Pasteurellaceae (family)	5	7.1	15.01
	Leptotrichiaceae (family)	4	5.7	15.46
	Proteobacteria (phylum)	4	5.7	17.29
	Gammaproteobacteria (class)	3	4.3	16.61
	Alcaligenaceae (family)	1	1.4	13.55
	Betaproteobacteria (class)	1	1.4	17.61
	Bifidobacteriaceae (family)	1	1.4	11.10
	Microbacteriaceae (family)	1	1.4	15.67
	Mycobacteriaceae (family)	1	1.4	11.85

Table 2. Loading values of Principal Component Analysis for 212 OTUs of total microbiome across 70 CF patients at stability. Large loading values are presented in bold (>0.0686). *AE=aerobe, facultative anaerobe, AN=obligate anaerobe, UNCL=unclassified. The first two principal components accounted for 91.44% of the variance (PC 1, 82.96% and PC 2, 8.48%)

*Classification	OTU	PC 1	PC 2
AE	*Staphylococcus sp.*	0.98809	0.008994
AE	*Staphylococcus aureus*	0.15247	-0.00441
AE	*Actinomyces odontolyticus*	0.012543	0.008639
AE	*Pseudomonas sp.*	0.005804	0.10697
AN	*Porphyromonas pasteri*	0.004126	0.011667
AN	*Prevotella melaninogenica*	0.002636	0.20915
UNCL	*Actinobacteria (class)*	0.002463	0.016196
AN	*Porphyromonas sp.*	0.001253	0.027859
AE	*Haemophilus sp.*	0.00041	-0.00023
AE	*Serratia sp.*	0.000246	-0.00043
AE	*Actinomyces graevenitzii*	0.000233	0.000741
AN	*Atopobium sp.*	0.000199	0.002323
AE	*Gemella sp.*	0.000191	0.013642
AE	*Campylobacter sp.*	7.77E-05	-3.95E-05
AE	*Campylobacter gracilis*	5.38E-05	0.0006
AE	*Haemophilus parahaemolyticus*	3.74E-05	-1.90E-05
AN	*Ihubacter sp.*	3.13E-05	0.004212
AN	*Prevotella intermedia*	2.57E-05	-1.31E-05
AE	*Actinomyces gerencseriae*	1.84E-05	0.000129
AN	*Tannerella forsythia*	1.48E-05	-2.65E-05
AN	*Atopobium sp. DMCT15023*	1.31E-05	0.002196
AN	*Peptococcus sp. oral taxon 167*	1.17E-05	-5.97E-06
AN	*Prevotella conceptionensis*	1.17E-05	3.73E-05
AE	*Neisseria sp.*	1.07E-05	-0.00013
AE	*Corynebacterium durum*	9.35E-06	2.77E-05
AE	*Streptobacillus hongkongensis*	8.80E-06	-4.48E-06
AE	*Pseudomonas sp. WHAS46*	8.31E-06	3.90E-06
AN	*Parvimonas micra*	7.16E-06	0.003635
AN	*Fusobacterium nucleatum*	4.08E-06	-0.00048
AN	*Treponema lecithinolyticum*	3.40E-06	0.00057

AE	*Staphylococcus sp. KSUM 23*	3.10E-06	-0.0002
AE	*Streptococcus australis*	-8.70E-08	-7.09E-07
AE	*Leptotrichia hofstadii*	-2.13E-07	-2.88E-06
AE	*Marinifilum sp. S1-C*	-2.40E-07	-1.94E-06
AE	*Streptococcus sp. C206*	-2.86E-07	-2.27E-06
AN	*Parvimonas sp. KA00067*	-3.33E-07	-2.16E-06
AE	*Corynebacterium sp. oral taxon B00*	-3.82E-07	-3.10E-06
AE	*Haemophilus sputorum*	-4.00E-07	-3.54E-06
AE	*Ancylomarina subtilis*	-4.20E-07	-3.40E-06
AE	*Streptococcus sp. oral taxon G62*	-6.56E-07	-1.25E-06
AN	*Treponema sp. OMZ 838*	-6.67E-07	-5.41E-06
AE	*Cardiobacterium hominis*	-6.97E-07	-4.86E-06
AN	*Porphyromonas gingivalis*	-7.17E-07	-5.00E-06
AE	*Gemella asaccharolytica*	-7.45E-07	-4.96E-06
AN	*Sneathia amnii*	-7.64E-07	-5.59E-06
AE	*Ottowia sp. Marseille-P4747*	-1.03E-06	-9.12E-06
AE	*Actinomyces sp. oral strain Hal-1065*	-1.14E-06	-9.27E-06
AE	*Sulfurihydrogenibium sp. T-7*	-1.23E-06	-9.42E-06
AE	*Haemophilus haemolyticus*	-1.29E-06	-7.52E-06
AN	*Prevotella sp. 8404125*	-1.33E-06	-8.63E-06
AE	*Cardiobacterium valvarum*	-1.34E-06	-9.32E-06
AE	*Pseudomonas sp. EJ01*	-1.38E-06	-8.55E-06
AE	*Streptococcus massiliensis*	-1.46E-06	-6.30E-06
AE	*Lachnobacterium sp.*	-1.54E-06	-1.18E-05
UNCL	*Bifidobacteriaceae (family)*	-1.56E-06	-9.18E-06
AN	*Solobacterium moorei*	-1.62E-06	-4.67E-06
AE	*Pseudomonas resinovorans*	-1.62E-06	-1.24E-05
AE	*Haemophilus sp. HFH0072*	-1.66E-06	-9.67E-06
AN	*Bifidobacterium breve*	-1.69E-06	-1.27E-05
AE	*Leptotrichia massiliensis*	-1.70E-06	-9.54E-06
AN	*Sneathia sanguinegens*	-1.70E-06	-1.13E-05
AN	*Anaerococcus obesiensis*	-1.71E-06	-9.56E-06
AN	*Prevotella sp. oral taxon 299*	-1.72E-06	-1.00E-05
AE	*Streptococcus sp. oral taxon 071*	-1.75E-06	-6.94E-06
AE	*Corynebacterium sp.*	-1.83E-06	-5.48E-06
AN	*Prevotella salivae*	-1.92E-06	1.43E-05
AE	*Pseudomonas otitidis*	-1.93E-06	-1.43E-05
AN	*Bacteroides fragilis*	-1.95E-06	-1.09E-05
AE	*Actinobaculum sp.*	-1.97E-06	-1.44E-05
AN	*Ruminiclostridium cellobioparum*	-2.07E-06	-1.58E-05
AN	*Oribacterium parvum*	-2.45E-06	-1.64E-05
AN	*Veillonella parvula*	-2.58E-06	-2.29E-05
AE	*Neisseria cinerea*	-2.60E-06	-2.30E-05
UNCL	*Mycobacteriaceae (family)*	-2.62E-06	-4.98E-06
AE	*Leptotrichia sp. oral taxon 212*	-2.66E-06	5.22E-05
AE	*Aggregatibacter aphrophilus*	-2.78E-06	-2.09E-05
AN	*Shuttleworthia sp. MSX8B*	-2.95E-06	-8.24E-06
AE	*Pseudomonas sp. P1-5*	-3.06E-06	-1.80E-05
AN	*Prevotella shahii*	-3.27E-06	-2.51E-05
AE	*Pseudomonas sp. 1W*	-3.48E-06	-2.59E-05
AE	*Janthinobacterium sp.*	-3.70E-06	-3.04E-05
AN	*Catonella morbi*	-4.23E-06	-2.37E-05
AE	*Leptotrichia hongkongensis*	-4.29E-06	0.000328
AN	*Fusobacterium periodonticum*	-4.34E-06	-3.14E-05
AE	*Actinomyces sp. ChDC B246-I*	-4.46E-06	-1.57E-05
AN	*Treponema maltophilum*	-4.84E-06	-9.19E-06
AE	*Gemella haemolysans*	-4.92E-06	4.58E-05
AE	*Streptococcus oralis*	-4.95E-06	8.94E-05
AN	*Fusobacterium necrophorum*	-5.11E-06	-3.40E-05
UNCL	*Enterobacterales (order)*	-5.38E-06	-4.47E-05

AE	*Bergeyella cardium*	-5.51E-06	-3.57E-05
AN	*Anaerococcus sp.*	-5.52E-06	4.91E-05
AN	*Slackia sp. CM382*	-5.63E-06	-3.15E-05
AE	*Arthrobacter sp.*	-6.18E-06	-5.06E-05
AE	*Gemella sanguinis*	-6.51E-06	-3.23E-05
AE	*Streptococcus constellatus*	-7.02E-06	-2.64E-05
AE	*Lawsonella clevelandensis*	-7.37E-06	-4.18E-05
AN	*Scardovia sp.*	-7.60E-06	-5.74E-05
AE	*Rothia dentocariosa*	-7.63E-06	0.000391
AE	*Streptococcus peroris*	-7.79E-06	-0.00012
AE	*Leptotrichia sp. PG10*	-8.20E-06	0.000161
AE	*Eubacterium sp. oral strain A35MT*	-8.30E-06	8.92E-06
UNCL	*Neisseriaceae (family)*	-8.80E-06	-5.83E-05
AE	*Yokenella sp.*	-8.89E-06	-3.53E-05
UNCL	*Alcaligenaceae (family)*	-8.90E-06	-9.90E-05
AE	*Pseudomonas sp. CFWTS4*	-8.95E-06	0.000321
AE	*Enterococcus faecium*	-9.07E-06	-6.84E-05
AN	*Olsenella sp.*	-9.45E-06	-2.71E-05
AE	*Kingella oralis*	-9.69E-06	-4.38E-05
AE	*Campylobacter concisus*	-9.71E-06	-5.76E-05
AE	*Capnocytophaga sp. S12-14*	-1.00E-05	-5.97E-05
AE	*Actinomyces sp. oral taxon 170*	-1.15E-05	-6.66E-05
AN	*Finegoldia sp.*	-1.27E-05	-3.29E-05
AE	*Lactobacillus sp.*	-1.33E-05	0.001357
AE	*Gemella sp. sp2-iso-ab31*	-1.34E-05	7.71E-05
AE	*Actinomyces oris*	-1.39E-05	-7.76E-05
AE	*Streptococcus pneumoniae*	-1.52E-05	-1.01E-05
AE	*Actinomyces sp. ICM54*	-1.62E-05	-0.00013
AN	*Prevotella sp. oral taxon 292*	-1.70E-05	1.84E-05
AE	*Actinomyces sp. ICM41*	-2.03E-05	-0.00011
AN	*Johnsonella sp. oral taxon 166*	-2.06E-05	2.24E-05
AE	*Leptotrichia genomo sp. C1*	-2.26E-05	-0.00014
AN	*Tannerella sp. oral taxon HOT-286*	-2.30E-05	0.000215
AN	*Peptoniphilus sp.*	-2.36E-05	0.001028
AE	*Streptococcus sp. 3192A*	-2.40E-05	0.000287
UNCL	*Pasteurellaceae (family)*	-2.43E-05	0.000161
AN	*Prevotella nigrescens*	-2.46E-05	-9.81E-05
AE	*Dysgonomonas sp. PH5-45*	-2.54E-05	-0.00017
AE	*Achromobacter xylosoxidans*	-2.72E-05	-0.0003
AN	*Veillonella atypica*	-2.85E-05	0.00206
AE	*Actinomyces sp. ICM39*	-2.95E-05	-8.80E-05
AN	*Megasphaera sp. sp4-iso-1H02x2*	-2.98E-05	0.002157
UNCL	*Leptotrichiaceae (family)*	-3.15E-05	-0.00018
AE	*Streptococcus cristatus*	-3.20E-05	0.002282
AE	*Gemella morbillorum*	-3.45E-05	0.002329
AN	*Porphyromonas endodontalis*	-3.76E-05	0.000911
AE	*Streptococcus sp.*	-3.79E-05	0.002153
AE	*Leptotrichia shahii*	-3.80E-05	0.00197
UNCL	*Microbacteriaceae (family)*	-3.80E-05	0.002114
AN	*Dialister sp.*	-3.83E-05	0.000722
AE	*Streptococcus salivarius*	-3.88E-05	0.002342
AE	*Actinomyces sp. oral taxon 848*	-3.99E-05	0.000769
AE	*Haemophilus parainfluenzae*	-4.12E-05	0.001703
AE	*Actinomyces sp. R42.11*	-4.25E-05	0.000297
AE	*Actinomyces lingnae*	-4.35E-05	4.19E-05
AN	*Shuttleworthia sp.*	-4.53E-05	0.002678
AN	*Mogibacterium sp.*	-4.61E-05	0.00121
AN	*Tannerella sp.*	-4.74E-05	0.000279
AN	*Porphyromonas catoniae*	-4.83E-05	0.000227
AN	*Alloprevotella tannerae*	-5.07E-05	-0.00011

AE	*Corynebacterium segmentosum*	-5.77E-05	6.25E-05
AN	*Peptostreptococcus anaerobius*	-6.03E-05	-0.00024
AE	*Streptococcus mitis*	-6.05E-05	0.002294
AE	*Enterococcus sp.*	-6.05E-05	-0.00045
AN	*Porphyromonas sp. C1075*	-6.30E-05	0.00073
UNCL	*Gammaproteobacteria (class)*	-7.03E-05	-0.00058
AE	*Actinomyces sp.*	-7.15E-05	0.000103
AN	*Lachnoanaerobaculum umeaense*	-7.27E-05	7.89E-05
AE	*Abiotrophia defectiva*	-7.36E-05	0.002527
AE	*Capnocytophaga sp.*	-7.43E-05	-0.00023
AE	*Lautropia sp.*	-8.74E-05	-0.00074
AE	*Actinomyces sp. oral taxon 448*	-9.00E-05	0.004427
AE	*Nocardia sp.*	-9.84E-05	-0.00056
AE	*Actinomyces naeslundii*	-0.0001	0.000834
AN	*Prevotella histicola*	-0.00011	-8.59E-06
UNCL	*Proteobacteria (phylum)*	-0.00012	-0.00086
AE	*Corynebacterium sp.*	-0.00012	0.003114
AE	*Leptotrichia wadei*	-0.00014	0.004733
UNCL	*Betaproteobacteria (class)*	-0.00015	-0.00164
AE	*Moraxella nonliquefaciens*	-0.00015	-0.00054
AE	*Streptococcus mutans*	-0.00016	0.004718
AN	*Oribacterium sp.*	-0.00016	0.006922
AE	*Ralstonia solanacearum*	-0.00017	0.002549
AN	*Catonella sp.*	-0.00017	0.004582
AN	*Prevotella nanceiensis*	-0.00019	0.00838
AE	*Streptococcus lactarius*	-0.0002	0.010243
UNCL	*Clostridiales (order)*	-0.00023	0.005578
AE	*Capnocytophaga sputigena*	-0.00023	0.013287
AN	*Prevotella denticola*	-0.00025	-0.0018
AN	*Veillonella sp.*	-0.00025	0.012255
AE	*Granulicatella elegans*	-0.00025	0.008917
AN	*Prevotella pallens*	-0.00034	0.005489
AE	*Mycoplasma salivarium*	-0.00035	0.00466
UNCL	*Firmicutes (phylum)*	-0.00037	0.011337
AE	*Actinomyces sp. 1AG30-1x10*	-0.00037	0.00955
AE	*Pseudomonas aeruginosa*	-0.00038	0.007318
AE	*Capnocytophaga granulosa*	-0.0004	0.006453
AN	*Peptostreptococcus sp.*	-0.00044	0.007665
AN	*Atopobium rimae*	-0.00046	0.019287
AE	*Capnocytophaga gingivalis*	-0.0005	0.009687
AE	*Flavobacterium sp.*	-0.0005	-0.0021
AE	*Leptotrichia sp.*	-0.00052	0.016637
AN	*Prevotella oulorum*	-0.00053	0.003611
AN	*Alloprevotella Prevotella sp. oral taxon 474*	-0.00055	0.01148
AE	*Streptococcus anginosus*	-0.00057	1.47E-05
AN	*Moryella indoligenes*	-0.00066	0.022092
AE	*Actinomyces sp. sp4-iso-1H01*	-0.00077	0.014828
AN	*Stomatobaculum longum*	-0.00084	0.032871
AN	*Bifidobacterium sp.*	-0.00099	0.071182
AE	*Actinomyces sp. sp2-iso-aAG3x2*	-0.00107	0.069833
AN	*Scardovia wiggsiae*	-0.0012	0.048081
AN	*Prevotella oris*	-0.00185	-0.00413
UNCL	*Streptococcaceae (family)*	-0.00238	0.08811
AE	*Actinomyces sp.*	-0.00267	0.097203
UNCL	*Bacteria (Kingdom)*	-0.00268	0.22573
UNCL	*Lactobacillales (order)*	-0.00277	0.074788
AE	*Achromobacter sp.*	-0.00367	-0.04245
AE	*Streptococcus sp.*	-0.00415	0.81534
UNCL	*Actinobacteria (phylum)*	-0.00491	0.28024

AE	*Rothia sp.*	-0.00521	0.21993
AE	*Rothia mucilaginosa*	-0.006	0.18357
AN	*Prevotella sp.*	-0.00728	0.16146

Table 3. The 10 most abundant obligate anaerobic bacteria identified at species level present in sputum at clinical stability (N=70).

Anaerobic species (OTUs)	n (%)	Range*
Prevotella melaninogenica	52 (74.3)	0 to 3.7 x 10^6
Scardovia wiggsiae	31 (44.3)	0 to 5.8 x 10^5
Porphyromonas pasteri	21 (30.0)	0 to 2.6 x 10^5
Parvimonas micra	15 (21.4)	0 to 2.5 x 10^5
Prevotella pallens	14 (20.0)	0 to 2.4 x 10^5
Stomatobaculum longum	9 (12.9)	0 to 6.6 x 10^5
Fusobacterium nucleatum	9 (12.9)	0 to 1.5 x 10^5
Prevotella nanceiensis	9 (12.9)	0 to 2.0 x 10^5
Prevotella oris	8 (11.4)	0 to 1.6 x 10^6
Atopobium rimae	7 (10)	0 to 4.5 x 10^5

n; number of participants in which the individual bacterial species was present
*; relative abundance before $\log_2$ transformation
